# Supplementary figures and images for: A qualitative study to understand public views on the relative value of health gains for children and young people in Australia compared to adults
Source: PLoS One. 2025 Oct 31;20(10):e0319227. doi: 10.1371/journal.pone.0319227 (PMC12578166; doi:10.1371/journal.pone.0319227)

## S1 File. Coding Tree

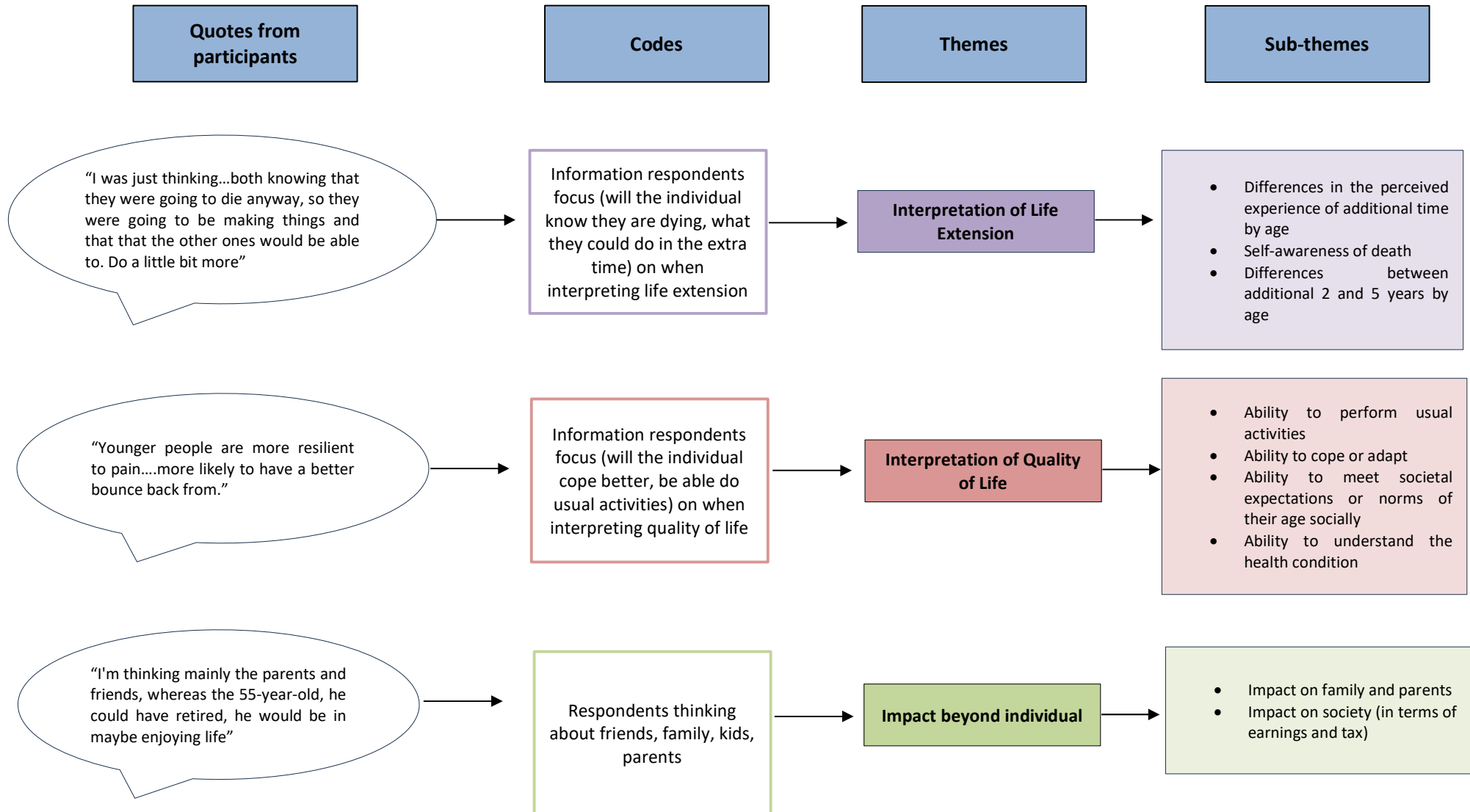

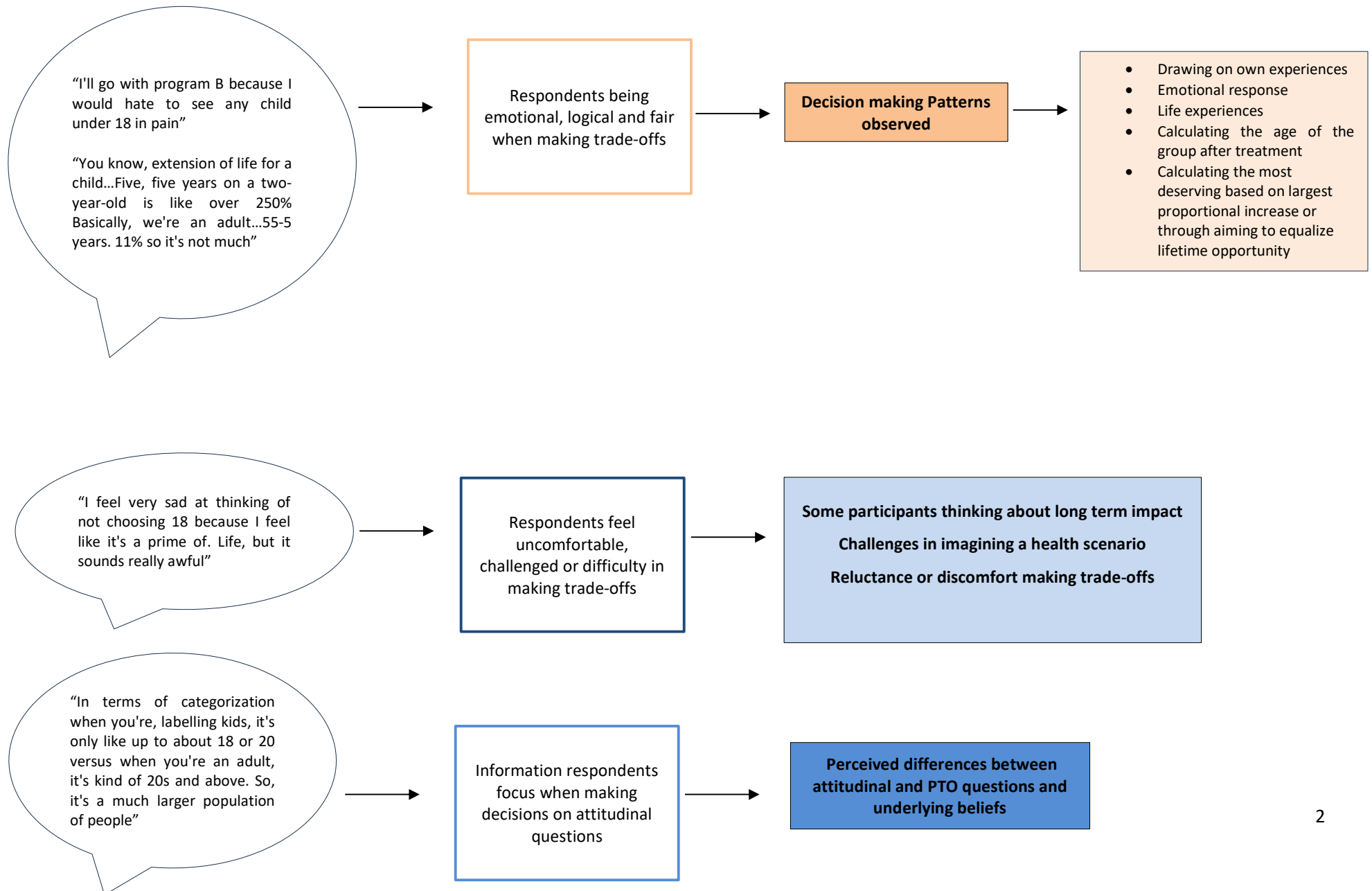

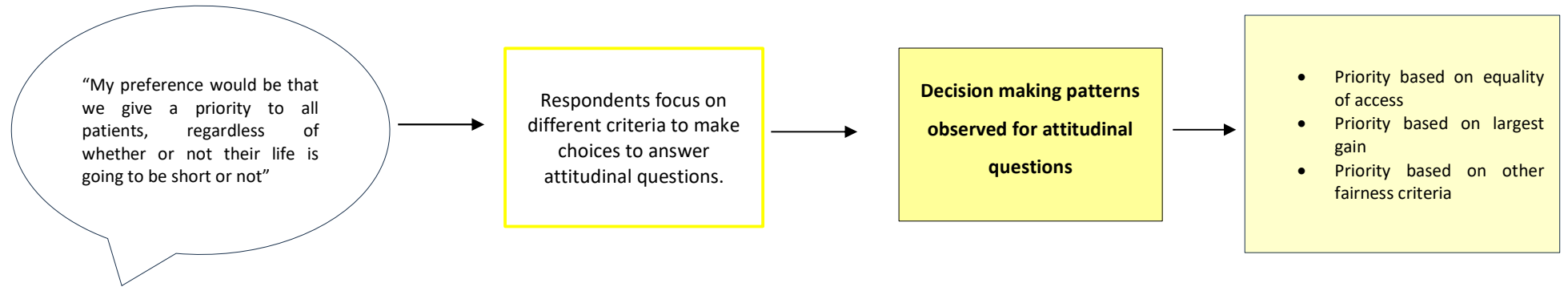

Supplement: S1 File — (PDF) [file pone.0319227.s001.pdf]
