# Supplementary material for: A qualitative study to understand public views on the relative value of health gains for children and young people in Australia compared to adults
Source: PLoS One. 2025 Oct 31;20(10):e0319227. doi: 10.1371/journal.pone.0319227 (PMC12578166; doi:10.1371/journal.pone.0319227)
Supplement: S2 File — (PDF) [file pone.0319227.s002.pdf]

Welcome to our value in child health study. Thank you for joining us. My name is TP. I'm going to tell you a bit about the questions in the survey and a bit about why we're doing this study.

This survey is all about prioritizing healthcare and we'd like you to imagine you've been asked to advise a healthcare decision maker, for example, someone working in government. Who has to make difficult choices about which treatments to fund. Often, they can't do everything they might like to do, and they have to prioritize between treatments and sometimes those treatments are for patients of different ages. Decision makers will want to consider the overall costs and benefits of new treatments, and they can measure improvements in the health of patients. They can also measure some other things that might differ, like the costs of treatments, the impact on the lives of caregivers who could be parents or spouses of the patient, for example. And they can also measure the impact on the patients or their caregivers ability to do their jobs. What they don't know, though, is whether people in society think there's something else which makes providing better health to different age patients more or less important is the same health gain more important or more valuable for patients of different ages? That's what we're trying to find out here.

In the survey, we're going to ask you questions where you choose between two treatment programs, imaginatively named A & B, and these treatment programs will differ across the questions, but the amount of health gain is always the same for patients in both A & B. We want you to imagine the decision makers have already found out that there's no difference overall in cost programs or the impact on carers or on people's income. So just for the sake of these questions, we want you to imagine the only difference is the age of the patients. Many of the questions look a bit like this. We want you to use only the information in the table to choose the program you think a decision maker should choose. Now some questions may show the option no preference. You can select this if you think the value of the two programs is the same and you just don't have a preference between funding either A or B. In other words, you'd be quite happy for the decision maker to make a decision based on the toss of a coin. Let's just break the table down a bit. The first row table shows us the patient's age at the start of treatment. The 2nd row shows us what the gain is from the treatment. So in this case the programs give the patients two more years of life, after which time they'll die. So those in program A would die age 62 and those in program B would die age 92. Now the last row shows us how many patients each program will be able to treat, and the questions always start at 100 people for both. But the number in the programs can change.

So, let's look at an example of this type of question. If I was doing this. I would have to think whether I should choose program A or B. Which is better which gives the health gain that I think is more important. I need to remember that the amount of health gain is exactly the same, and we're assuming

that the overall cost of the programs is the same. The impact on the carers and any loss in income is the same. The only difference is the age of the patients. Let's imagine I choose program A, so I select the button underneath that column, but of course you might have a different view. The next question looks very similar, but the number of patients in program A has now changed to 50. And I have to choose again. In some of the questions, the health gain is avoiding a temporary illness for two years. In this case, it's avoiding an illness which would have caused problems walking or moving. For these questions, we assume that after the two years, all the patients would return to normal health with no long term consequences. We can also ask you some questions to help us understand why you answered the way you did and to understand your views on prioritizing healthcare. Now there are no right or wrong answers here, we just want to know your opinions. We think these questions are quite difficult and we also appreciate that for some people they might be quite close to some emotionally very sensitive issues. So we do think it's important we explain why we're asking. Decision makers that are responsible for the Medicare budget in Australia make decisions about how to spend that budget and if they're considering buying a new drug for children, for example, it will be useful for them to know whether the Australian public thinks their health benefits are more or less important, if they're for children, this will help them know whether they should be willing to pay more for new treatments for children. And we'll be sharing our findings with decision makers. So thank you very much for listening and thank you for sharing your views with us. Let's get started.
