# Supplementary material for: A qualitative study to understand public views on the relative value of health gains for children and young people in Australia compared to adults
Source: PLoS One. 2025 Oct 31;20(10):e0319227. doi: 10.1371/journal.pone.0319227 (PMC12578166; doi:10.1371/journal.pone.0319227)
Supplement: S1 Table — (DOCX) [file pone.0319227.s003.docx]

**S1 Table. Characteristics of study participants**

|  | Number/ Proportion |
| --- | --- |
| N | 41 |
| Female | 23 (56%) |
| Age Distribution |  |
| 16-18 years | 7 |
| 19-55 years | 24 |
| ≥ 55 years | 10 |
| Adults with no children | 11 |
| Child(ren) have experienced a serious illness |  |
| Yes | 15 |
| No | 8 |
| Adolescents (16-18 years) | 7 |
| Employment status  Employed or self-employed-Full time | 14 |
| Employed or self-employed-Part time | 10 |
| Student | 9 |
| Retired | 5 |
| Maternity Leave | 1 |
| Not working due to poor health | 1 |
| Not working due to caring responsibility | 1 |
| Country of birth |  |
| Australia | 31 |
| Elsewhere | 10 |
|  |  |
