# Supplementary material for: A qualitative study to understand public views on the relative value of health gains for children and young people in Australia compared to adults
Source: PLoS One. 2025 Oct 31;20(10):e0319227. doi: 10.1371/journal.pone.0319227 (PMC12578166; doi:10.1371/journal.pone.0319227)
Supplement: S2 Table — (DOCX) [file pone.0319227.s004.docx]

**S2 Table. Differences in participants characteristics between recruitment methods**

|  | Number | |
| --- | --- | --- |
|  | **CRNRSTONE Sample** | **P-MIC Sample** |
| N | 26 | 15 |
| Female | 11 | 12 |
| Age Distribution  16-18 years  19-55 years  ≥ 55 years | 7  9  10 | 0  15  0 |
| Adults with no children  Child(ren) have experienced a serious illness   - Yes - No   Adolescents (16-18 years) | 11  2  6  7 | 0  13  2  0 |
| Employment Status  Employed/ self-employed  Student  Retired  Maternity leave  Not working | 12  9  5  0  0 | 12  0  0  1  2 |
