# Supplementary material for: A qualitative study to understand public views on the relative value of health gains for children and young people in Australia compared to adults
Source: PLoS One. 2025 Oct 31;20(10):e0319227. doi: 10.1371/journal.pone.0319227 (PMC12578166; doi:10.1371/journal.pone.0319227)
Supplement: S3 Table — (DOCX) [file pone.0319227.s005.docx]

**S3 Table. Additional quotes from participants**

| **Theme 1: Interpretation of Life Extension** | |
| --- | --- |
| 1.1 Differences in the perceived experience of additional time by age | *“there's more opportunities for them to do something with those two years of substance than a 10 year old”* [Male,37yrs: PTO 10 yrs/20 yrs]  *“You know, at the age of 20, you’re just becoming an adult. You've got a lot of big things ahead of you. You might have a partner that you want to get married to, you might have that partner you might want to have kids”* [Male,19yrs: PTO 20 yrs/40 yrs]  *“I think program A because 4-year-olds haven't really experienced life then, even as like a five- or six-year-old, they weren't really remember so….like a 14 year old. I guess they have a lot more wider like friends and family, and I think it will impact them more and will benefit them more if they live longer because then they can spend that time with friends and family”* [Female,16yrs: PTO 14 yrs/4 yrs]  *“Immediately my mind goes to program B (10 yrs) with the younger patient just for the fact that they are more likely to have experienced less in their time and I guess tick less things off their bucket list”* [Male,19yrs: PTO: 10 yrs/ 40 yrs]  *“I would lean more towards program B, for the age of 14 year olds, because you know…these teenagers are still quite young, it gives them the opportunity to live life to the fullest. They haven't been around for very long”* [Male,27yrs: PTO 14 yrs/40 yrs]  *“I'd be just - 2 years of a 12-year-old just feels like they would experience more and that's like…you know, the transition starting to adulthood I feel like, that's a, not a bigger treatment but a bigger part of life”* [Male,39yrs: PTO 12 yrs/2 yrs]  *“For me, allowing five years onto the 16-year-olds, they can go to the age of 21, which means they can go through and get a degree and be ready to start that life and be able to experience the joys of youth going out to clubbing in the valley and stuff like that. So, I would still give it to 16-year-olds in that situation”* [Male,19yrs: PTO 16 yrs/55 yrs] |
| 1.2 Self-awareness of death | *“I almost want to switch camps this time just based on how it would affect a 10 year old’s life knowing that you've got this treatment, but you're still going to die in two years’ time. I think that would be incredibly hard for a 10-year-old to stomach”* [Female,37yrs: PTO 10 yrs/55 yrs]  *“I thought, well, these people don't know this anyway, but if they knew. They would have a lot of things they would be able to do within that five years”* [Female,86yrs: PTO 24 yrs/55 yrs]  *“I feel like an 8-year-old doesn't have like such an understand. The 8-year-old doesn't have like an understanding of what it all means, where it would like impact. Like giving an extra two life two years of life to an 18-year-old would mean a lot more”* [Female,16yrs: PTO 18 yrs/8 yrs]  *“Now that's a really hard thing to choose because they're both children. Feel like a 10 year old would understand, so that would be quite scary for the child”* [Female,44yrs: PTO 1 month/10 yrs]  *“I think I would go with a 14-year-old just because they have more knowledge of life”* [Female,37yrs: PTO 14 yrs/ 4 yrs] |
| 1.3 Differences between an additional 2 and 5 years by age | *“I think just that now we're looking at five years and we're looking at kids from age 8 to 13. And I think that extending life by five years to me seems to be I've got more empathy for that age group”* [Male,75yrs: PTO 40 yrs/8 yrs]  *“I would still see that the age group of 2-7 so giving an additional five years to the two year old kid. I'd have seen like kids probably don't remember much, at least till the age of four or five. So I would definitely give additional five years to the 40-year group”* [Male,40yrs: PTO 2 yrs/40 yrs]  *“like 5 years you can actually accomplish something and like have something different. Two years is a bit shorter where, but like you can't really make that big of a change”* [Female,17yrs: PTO 40 yrs/14 yrs] |
| **Theme 2: Interpretation of Quality of Life** | |
| 2.1 Ability to perform usual activities | *“like 2 years can go fast or slow. But I think as a teenager I guess those years like 14/15/16 is where …like you grow a lot more. And I think like it's. important then to like have good like mental health because there's so many like challenges at school and stuff”* [Female,16yrs: PTO 14 yrs/40 yrs]  *“Well, like a 55-year-old if they can't move or have very problems moving well, that's a lot of effort to move a 55-year-old…there's only few 2-year-olds you can't pick up”* [Male,39yrs: PTO 55 yrs/2 yrs]  *“Because at 40 years of age, I think those patients would be in the prime of their life as far as their work is concerned, and they need more to be fit and healthy as far as walking and moving around is concerned, so I think. I think program A would be more beneficial to 40 year olds than eight year olds”* [Male,75yrs: PTO 40 yrs/8 yrs]  *“also, a 10-year-old, they're moving around a lot. They need to do exercise like you know, school and everything, it's. like with sport at school and things”* [Female,18yrs: PTO 10 yrs/40 yrs] |
| 2.2 Ability to cope or adapt | *“whereas a 1 month old hasn't learned it to begin with. So they're not going through those same struggles as what a 55-year-old would have to*” [Female,39yrs: PTO one month/55 yrs]  *“In program A age of patients of 40. So, by that time I think people are more mentally strong so they can handle themselves emotionally with the anxiety, but at the age of four it's very new in life. And eventually anyways, this is not life threatening. Yeah, I would encourage the younger patients to be treated well”* [Male,35yr:] PTO 4 yrs/40 yrs]  *“at the age of 18 they're probably less equipped with life experience to be able to navigate mental health issues or concerns or challenges”* [Female,42yrs: PTO 18 yrs/40 yrs]  *“I'm going to go A (18 yrs) because I feel like as an 18-year-old, living with a physical illness like that has pain that can be very isolating, and that that would be…really challenging as a young person”* [Female,42yrs: PTO 18 yrs/40 yrs]  *“I think that young people are more aware of the support that they have access to. I think they're more aware of mental health issues in general. Whereas someone that's a little bit older may not know how to access those resources and how to deal with appropriately”* [Male,19yrs: PTO 40 yrs/20 yrs]  *“When I was in and out of hospital a lot when I was a child, I felt it's I'm not saying that people in their 50s deserve to go through pain, but I feel that they're more better equipped to cope with pain, physical pain than a child and I think”* [Male,27yrs: PTO 8 yrs/55 yrs]  *“I'd probably pick program A cause. When you're older, you have weaker bodies and like your joints and burns will get weaker so pain would be more like severe compared to someone who's 14, who has a younger body and they're body can combat stuff easier than someone who's older”* [Female,17yrs: PTO 40 yrs/14 yrs]  *“I'm thinking that a 55 can probably take a pain a bit more longer than a bit better than a 24 so I would give the treatment to the 24”* [Female,86yrs: PTO 24 yrs/55 yrs]  *“I'm going with the 10 year olds because I think 55-year-olds can be more could generally be more resilient to pain”* [Male,57yrs: PTO 10 yrs/ 55 yrs]  *“Most, 55 or most adults I know sort of deal with, you know, levels of low mood and anxiety. I think it's just part of being an adult sometimes that you have…you just learn to cope and you learn to deal with it. And I think that you know if I had the choice between you know, being able to relieve my low mood or anxiety versus relieving that of the of the child. I'm gonna take the child every time. Because I've learned, I've got coping mechanisms that…helped me get through that”* [Male,57yrs: PTO 10 yrs/55 yrs]  *“Probably the 20-year-olds. It would be a massive barrier to them too, and that they would be more likely to struggle and overcome the pain. 40 year olds- you've had probably some degree of pain or suffering before you're 40, and yes, it's horrible, but you can adapt because you have longer to learn how to adapt”* [Female,40yrs: PTO 20 yrs/40 yrs] |
| 2.3 Ability to meet societal expectations or norms of their age socially | *“I guess these people are a bit more ingrained in society. And so, you know, they might have jobs they might have friends, they'll have all sorts of things. And…them feeling this two-year illness is probably worse for them than it would be for the four-year-old”* [Male,27yrs: PTO 40 yrs/4 yrs]  *“I'd definitely go with the 24. The 24 got more to loose…everything like social, the social stuff is big now with them and I think they would suffer more”* [Female,86yrs: PTO: 24 yrs/55 yrs] |
| 2.4 Ability to understand the health condition | *“program A 8-year-olds. With problems walking or moving, don't understand and I imagine there'd probably be something painful there. Whereas the 40-year-olds would understand what's going on”* [Female,75yrs: PTO 8 yrs/40 yrs]  *“the age of the patient would help them have a better understanding of what they're going through”* [Male,19yrs: PTO 55 yrs/12 yrs]  *“I think program A (8 yrs), children in pain, it's not a good thing. Adults-the 40-year-old understands pain”* [Female,75yrs: PTO 8 yrs/40 yrs]  *“he's (2 yrs) going to struggle more because he's not gonna realise what's happening to him”* [Male,77yrs: PTO 2 yrs/55 yrs]  *“Pain is very hard to explain to a child why they're experiencing it, where 55-year-old can explain what illness they have…but they can kind of have a logic behind it”* [Male,39yrs: PTO 2 yrs/55 yrs]  *“Treat the babies I think as an adult we've got a lot better understanding of pain. We've got better ways to manage it, you know, one month old, they don't. They don't understand pain. All they know is they're hurt and they're scared, which again leads on to that….trauma later in life and it's got a massive flow on effects, whereas the 55-year-old…you understand pain”* [Female,37yrs: PTO one month old/55 yrs]  *"I think I'll go program B because they are 4-year-olds…They don't remember much. They are still only developing”* [Male,19yrs: PTO 4 yrs/55 yrs] |
| **Theme 3: Impact beyond individual** | |
| 3.1 Impact on family and parents | *“Yeah, I'd probably go program A again, the 40-year-olds. Influenced being a parent and just thinking of the value of parents and their children”* [Female,34yrs: PTO 40 yrs/18 yrs]  *“I think a 12-year-old giving two extra years, especially for the family they would really kind of appreciate compared to a 55-year-old who's lived 55 years”* [Male,39yr: PTO 12 yrs/55 yrs]  *“five years to the 40-year-old could make a difference if they've got a young family”* [Female,75yrs: PTO 40 yrs/8 yrs]  *“So, let's just say the typical 40-year-old is meant to have settled down with the family and has kids but a six-year-old also has a family they've got parents as well. I think that perhaps program A because I think that 40-year-olds have a little bit more to live for”* [Female,18yrs: PTO 40 yrs/6 yrs]  *“I would probably choose program A (40 yrs). Just in terms of the effect on the family, yeah, thinking that they have children”* [Female,38yrs: PTO: 40 yrs/ 8 yrs]  *“I would still, as a parent, want that extra two years to prep and to grow my children”* [Female,32yrs: PTO 40 yrs/14 yrs]  *“I would go for the four year olds over the 40 because. I think in this situation I'm thinking more of the welfare of the parents and, you know, the people around these toddlers”* [Male,27yrs: PTO 4 yrs/40 yrs]  *“His parents and friends have had him for 12 years…The parents of the two year old have only had him for two years. I think they would like him to have him for another two years”* [Male,77yrs: PTO 2 yrs/12 yrs]  *“Giving younger, I guess…More time with family”* [Female,42yrs: PTO 8 yrs/ 18 yrs]  *“a parent losing a 2-year-old if they can get another five years, you know, feels like that's a great gain”* [Male,39yrs: PTO 2 yrs/55 yrs]  *“I have also known people who have had children who have passed away, quite young and then, you know, going through my head then would be. Knowing that you are, you've still got a, basically a date that it's only gonna help so much. Is it something that you would still do knowing that you're only buying a little bit of time and for those parents that I know who, children who have passed, they would have 100% said yes, absolutely. You know, anytime is better than no time. So based on that I would say the one-month-old”* [Female,37yrs: PTO one month/55 yrs]  *“Most people would want to give a 10-year-old, a little bit more chance and most families would want to keep their children with them”* [Female,37yrs: PTO 10 yrs/55 yrs] |
| 3.2 Impact on society (in terms of earnings, tax) | *“It sounds horrible to say, but I feel like it may be benefiting society a little bit more than a six-year-old”* [Female,18yrs: PTO 40 yrs/6 yrs]  *“There's more value for community in it. I mean, the 40 year old probably is a bit more productive than the 2 year old”* [Male,86yrs: PTO 40 yrs/2 yrs]  *“55 years patient has been giving out a lot of the activity to the society”* [Female,50yrs: PTO 55 yrs/4 yrs]  *“Most likely they (40 yrs) would also be having an impact on society in terms of work, so being able to contribute”* [Female,38yrs: PTO 40 yrs/ 18 yrs] |
| **Theme 4: Decision making Patterns Observed** | |
| 4.1 Drawing on own Experiences | *“I have a child who very much struggles with things that we take for granted every single day and he didn't choose any of it. And I know how hard it is for him and I know how uncertain things are for him and see if I knew that he was gonna get two years of a much better quality of life. And if he's gonna die, I would still choose. That, yeah. Because the quality of life and that processing time literally every minute. Counts when you're talking about your child's and I'm not a decision maker.* [Female,40yrs: PTO 10 yrs vs 40 yrs]  *“I'm going to choose Program B (12 yrs) just because of my own personal experience with like anxiety and mood, and I don't know how that would affect someone at 55, but I know how to affect someone at 12”* [Male,19yrs: PTO 12 yrs/55 yrs]  *“I look at my own self. I had a brain injury and a few strokes at age 50, which was a few years ago and received really good treatment and all that kind of stuff… Yeah, it was really helpful for me, and definitely extended my life”* [Male,57yrs: PTO 55 yrs/20 yrs]  *“I mean, yeah, I had a friend pass away from cancer a year and a half ago and yet she was in her 30s, and so I'm just thinking, you know, even if there was 50 families like her could have an extra 2 years of life”* [Female,38yrs: PTO 40 yrs/18 yrs] |
| 4.2 Emotional response | *“cruel to see a child die and I mean any child. I think it's just the mentality and I know it doesn't really feel rational, but it's still the thought”* [Female,38yrs: PTO 8 yrs/18 yrs]  *“I think now. I'm starting to get a bit sympathetic towards the 8-year-old children”* [Male,75yrs: PTO 8 yrs/18 yrs]  *“I think I'll go with program B (8 yrs) I've just, got more empathy for 8-year-old patient”* [Male,75yrs: PTO 8 yrs/40 yrs]  *“I had an amazing teacher who said no parent should see their child passed before them and I felt like that really stuck with me. After all these years, because I can only imagine the sort of sort of range of emotions that you feel through sadness and guilt”* [Male,27yrs: PTO 14 yrs/40 yrs]  *“I'll go with program B because I would hate to see any child under 18 in pain”* [Male,65yrs: PTO 12 yrs/40 yrs]  *“Cruel to see a child die and I mean any child”* [Female,38 yrs: PTO 8 yrs/18 yrs]  *“I'm saving the children, I'm always going to save the children”* [Female,45yrs: PTO 4 yrs/14 yrs] |
| 4.3 Life experiences | *“I would still go with program A (14 years) because I feel like for me, I think like 40 and 45, there's not really much difference between them where it's like from 14 to 19, I guess you have a gap because people I guess like consumed by like school and everything. But as you like move past like 18 you can do more stuff. You're more independent whereas it sort of feels like 40 and 45 is still pretty similar”* [Female,16yrs: PTO 14 yrs/40 yrs] |
| 4.4 Calculating the age of the group after treatment | *“Like 4- 6-year-olds…They don't really do much except like play. With like 40 year olds…they have a wide group of friends and family they have. Yeah, I think it's more like the social thing that they have more people around and they like, remember stuff”* [Female,16yrs: PTO 40 yrs/4 yrs]  *“I'm just sort of thinking a four-year-old then dies at 9, which is horrific, but a 40-year-old dying at 45, still horrific”* [Female,37yrs: PTO 4 yrs/40 yrs] |
| 4.5 Calculating the most deserving based on largest proportional increase or through aiming to equalize lifetime opportunity | *“You know what they could do in those five years and how much that five is represented in 10-year-olds effectively 50% of their life, seems more significant there”* [Male,37yrs: PTO 10 yrs/55 yrs]  *“2 years out of 55 is rather small. Whilst you know putting a 10 year old through 1/5 of his life in pain, could, you know, be rather traumatic, there might not be well equipped to kind of deal with the consequences”* [Male,37yrs: PTO 10 yrs/55 yrs]  *“40 years is a long time, and you know, I imagine that those people would have had a substantial ability to, you know, experience life, whether that be through, you know, getting married, finding love, having children, all those different sort of life stages”* [Male,27yrs: PTO 14 yrs/40 yrs] |
| **Theme 5: Some respondents thinking about long term impact** | *“But I think I'd have to prioritize program A (55 years) because I think the long-term impacts would be worse for someone at 55”* [Male,41yrs: PTO 55 yrs/8 yrs]  *“I don't want to traumatize them because if they go with a bunch of pain while they're young…it's not good. It's going to screw them up a little bit”* [Male,19yrs: PTO 16 yrs/55 yrs]  *“I know it says no long term health consequence, but I don't know if that considers everything holistically”* [Female,38yrs; PTO 18 yrs/40 yrs] |
| **Theme 6: Challenges in imagining a health scenario** | *“I don't feel like a baby at one month old could have a mental health illness”* [Female,44yrs: PTO 55 yrs/one-month]  *“Is that just pain in general”* [Female,16yrs: PTO 14 yrs/40 yrs]  *“the 40-year-olds have experienced the pain beforehand? Or like did it just start when they were 40?”* [Female,16yrs: one month/40 yrs]  *“I would like to know what's the intensity of the pain here. Are we talking about extreme pain, variable pain, minimal pain?”* [Male,35yrs: PTO 40 yrs/4 yrs]  “Like realistically, how would you actually witness that in a one month old as well? Like how? Could you even tell that they're going through, like, low mood and anxiety and stuff like that?” [Female,39yrs: PTO 55 yrs/one month] |
| **Theme 7: Reluctance or discomfort making trade-offs** | *“Not a happy camper making these choices”* [Female,42yrs: PTO 40 yrs/one-month]  *“I mean, how can you really prioritize one life over another? I mean, I've had little nieces and nephews they are all much older and I would not want it on me to prioritize one over the other”* [Female,37yrs: PTO one-month/10 yrs]  *“I feel very sad at thinking of not choosing 18 because I feel like it's a prime of life, but in terms of sounds really awful”* [Female,38yrs: PTO 40 yrs/18 yrs] |
| **Theme 8: Perceived differences between attitudinal and PTO questions and underlying beliefs** | *“tax rich people more tax mining billionaires* *and find the money to treat the extra people”* [Male,67yrs]  *“if it was like 40-year-olds versus 16-year-olds, for me that's kind of even… 40-year-olds aren't old, just like 16-year-olds aren't super young. To me like a 5-year-old might be” [Female,18yrs]*  *“we've only got an age group of under 18 which is compared to adults. So, the information is not as specific as choosing between A&B”* [Male,77yrs] |
| **Theme 9: Decision making patterns observed for attitudinal questions** | |
| 9.1 Priority based on Equality of access | *“Give the same priority to treating all patients. Australian Medicare it's for everybody”* [Female,75yrs]  *“I think Medicare to give the same priority”* [Male,77yrs]  *“Same priority to treating patients. I'm sick so treat me the same as anyone else who's sick”* [Female,86yrs]  *“founding value of what Medicare is that it's around treating people fairly and equally”* [Male,27yrs] |
| 9.2 Priority based on largest gain | *“Medicare should give priority to treating patients who will die young”* [Male,40yrs] |
| 9.3 Priority based on other fairness criteria | *“give priority of triggering patients who will get the lowest amount of health benefit”* [Male,19yrs]  *“You fund not those based on how old they are, but based on who can afford the treatment, someone who is otherwise wealthy can pay for a private treatment that the government doesn't need to support them. It doesn't matter whether they're old or young. This is about funding. You fund those who can't otherwise fund it”* [Male,36yrs]  *“Medicare should give priority to treating patients who would get the largest amount of health benefits from treatment”* [Male,19yrs]  *“I don't think it should get priority to patients who would die young unless you can guarantee positive outcomes”* [Male,39yrs]  *“I think the best we can do is to base it on a combination of who will get the best benefit and who will have longevity”* [Female,37yrs]  *“just whether or not the treatments going to lead to an improvement in health or lots of years of extra life”* [Female,50yrs] |
