# Supplementary material for: A qualitative study to understand public views on the relative value of health gains for children and young people in Australia compared to adults
Source: PLoS One. 2025 Oct 31;20(10):e0319227. doi: 10.1371/journal.pone.0319227 (PMC12578166; doi:10.1371/journal.pone.0319227)
Supplement: S3 File — (PDF) [file pone.0319227.s007.pdf]

Consent Form

Valuing health care for children, adolescents and young adults compared to valuing health care for adults

Thank you for taking the time to complete this survey.

During this survey you will be asked to make some choices between different patients and treatment programs (they are all hypothetical - in other words we made them up just for this task). We will then ask some background questions about you and your health.

The survey will take about 50 minutes to complete.

Why are we asking these questions? We are trying to find out whether people feel differently about the government funding health treatments which improve the health or length of life of children compared to adults. The findings from this study will be shared with decision makers in government to better inform decision makers about what you think is the best way to use healthcare funds.

What are the possible risks? Although we expect there are no risks, thinking about children leading short lives or lives with poor health may cause distress to some people. Your participation is voluntary. You can withdraw from this research at any time and without any consequences. All information you provide is confidential and will not be used in any way that could identify you.

You can find out more details about this study in this document [click here]. This document gives some additional information such as who is involved in the research, who is funding the research, and how we will use and store your data. If you would like to find out more about the study before you complete it, or ask questions, then please email: XXX

We would like to know that you are happy to take part the study.

Please click if you agree with each statement:

- ☐ I consent to participate in this research, the details of which have been explained to me
- ☐ I understand that my data may be shared with other researchers (but only anonymised data or in a way that I couldn't be identified)
