# Supplementary material for: A qualitative study to understand public views on the relative value of health gains for children and young people in Australia compared to adults
Source: PLoS One. 2025 Oct 31;20(10):e0319227. doi: 10.1371/journal.pone.0319227 (PMC12578166; doi:10.1371/journal.pone.0319227)
Supplement: Attachment — Submitted filename: Response to Editor.docx [file pone.0319227.s011.docx]

**Response to Editor**

Dear Adrian Cyrus Luczon,

I sincerely appreciate your thoughtful comments and feedback.

The data included currently within the manuscript and the Supporting Information Files (Quotes from participants, the coding tree, demographic characteristics) would constitute the "minimal data set". We have now included additional quotes in the supplementary material.
