# Supplementary material for: A qualitative study to understand public views on the relative value of health gains for children and young people in Australia compared to adults
Source: PLoS One. 2025 Oct 31;20(10):e0319227. doi: 10.1371/journal.pone.0319227 (PMC12578166; doi:10.1371/journal.pone.0319227)
Supplement: Attachment — Submitted filename: comments for minor revision.docx [file pone.0319227.s009.docx]

## **Title:** The title precisely reflects the subject of the study; however, it is academically advantageous to consider substituting the term "people" with "public."

**“A qualitative study to understand public views on the relative value of health gains for children and young people compared to adults”**

**Abstract:**

- **Methods:** Concise overview. Nonetheless, I suggest the inclusion of the following statement at the last line of the methods used paragraph: **“Thematic analysis was employed to identify fundamental reasoning patterns.”**

**Introduction:**

The introduction is thorough and based in robust research. However, Structural enhancements are necessary to improve the clarity and scholarly tone, such as:

- **Lines (108 –128), (129-142),** The paragraphs are too dense and long, consider **break them into clear paragraphs.**
- **Line 113,** those who have not had their fair innings **needs** to be prioritized. Change needs to **“need”**
- **Line 120,** consider changing **>5 years** to **“older than 5 years”** for more academic tone.

**Methods:** Demonstrate methodological transparency, provide a strong rationale for recruitment, and offer clear justification for the selections. Nevertheless, it requires slight modifications for improved clarity, formatting, and organization.

- **Recruitment and Participants:** Lines (164-179) and (180-192) consist of extensive paragraphs; **please divide them into shorter paragraphs.**
- **Survey Design:** The paragraph is extensive, consisting of lines 198-210. It is desirable to **divide it into two paragraphs.**
- **Data Collection:** The paragraphs lines (218-227) and (233-253) are extensive; therefore, it is necessary to **divide them into separate paragraphs.**

**Results:**

- **Participants section:** It is well written; however, **please divide it into paragraphs** for enhanced readability.
- **Qualitative Results:** it is well organized and comprehensive, but consider this:
  a) rewrite the sentence “Another participant chose the 40-year-olds over the 8-year-olds” in the line 333, for more academic tone **“participants aged 40 over those aged 8”**
  b) line 337 change the statement “people who are younger have to walk a lot more than someone who's 40” to **“Younger participants were viewed as more reliant on physical mobility, compared to those who are 40 years old”**
  c) Theme 5: Provide a short analytical statement elucidating the reason for this and why it happened for instance, **“Despite the guidance, participants struggled to disengage from real-world connections to chronic illness, illustrating the psychological challenge of ‘bracketing’ prolonged considerations.”**

**Discussion:** This discussion is substantial and effective, but it can be enhanced through refinement in:

- lines (535-554) and (637-662), are lengthy, **please divide them into paragraphs.**
- **Line 566-571** showing your **novel contribution** to the literature and the readers need to see this, so please add a statement that clarify this in the **line 571** such as **“To our knowledge, this is the first study to document………”**
- Line 542, change the informal statements please “unable to imagine” to **“had difficulty conceptualizing”**

**Conclusion:** It offers clear alignment with study objectives, acknowledges cognitive and emotional complexity, and is relevant to policy and methodology. However, please consider some amendments such as:

- Line 674, These interpretations were reasoned by factors such as… change to These interpretations were **influenced** by factors such as… (for an elevated research tone).
- Replace informal language with academic ones such as line 671 “drawing on emotional reaction” to **“triggering emotional response”,** and line 676 “what they might experience if they live” to **“anticipated life experiences”**
- Add word “**Additionally**” before (4) When subjected to alternative viewpoints… (To enhance the coherence between sentences for improved readability).
- The conclusion indicates that **four out of five aims** were addressed; however, it does not provide **a brief explanation** for the aim that was not addressed. A brief mention would enhance transparency.
- The final sentence suggests practical relevance; however, the conclusion would be enhanced by adding a **strong closing statement** regarding the implications of the findings for healthcare policymakers, PTO methodology, or valuation research. Please consider adding this.
