# Supplementary material for: A qualitative study to understand public views on the relative value of health gains for children and young people in Australia compared to adults
Source: PLoS One. 2025 Oct 31;20(10):e0319227. doi: 10.1371/journal.pone.0319227 (PMC12578166; doi:10.1371/journal.pone.0319227)
Supplement: Attachment — Submitted filename: N Kone_PLOS reviewer comments Sept. 2025.docx [file pone.0319227.s010.docx]

**NK**- PLOS reviewer comments on **“A qualitative study to understand people’s views on the relative value of health gains for children and young people compared to adults”** publication.

Thank you for giving me the opportunity to review the article mentioned above.

I can see that previous reviewers have given fair remarks, to which I would like to add the following:

**Abstract**

- The objective**s** should be replaced by aim of the study, there is only 1 (if it was to be objective) mentioned, which reflects the aim of the study (line154) and the 5 objectives later (lines156-161)

**Introduction:**

- Line 124 mentions “studies which found…”, yet there is only 1 reference (line125)
- Line 148: “Key evidence…” – key should be in small letters

**Methods**

- **Recruitment and Participants:** Line 167 – add the full range age of participants (16- xxx years), which is not the same as the target groups (e.g 2 years), because this latter group was not interviewed and didn’t contribute to the findings.
- **Survey Design:** Lines 202-203 (*13 age categories*) – please justify/explain choices for categories
- **Data Collection:**
  - Line 234: example?
  - Line 241: that example could be provided (even if the video is not for public consumption)
  - Line 250-251: please clarify…sentence is not clear
  - Line 252: reads better as "Interviews were video-recorded and transcribed using automated intelligent verbatim transcription."
- **Data Analysis**
  - Line 258: an important key demographic to consider was employment/unemployment – but in the manuscript, it is only mentioned later in the results section.

**Results:**

- **Participants**
  - Line 264 – recruitment was done over a period of four months (27^th^ March- 20^th^ July 2023)
- **Qualitative results:**
  - Table 1: 4.1 and 4.2 – small letters
- The authors should try as much as possible to replace words like *many (lines 341, 523), few (line 352, 525)*, etc…by numbers/percentage (quantities) like you have done in other instances.
- Line 390: What was program A -has it been defined/mentioned previously? (same as program B – line 397)
- Lines 572 and 580 – nice to see a number/percentage attached to the participants category, would like to see the same on line 579.
- Line 633 – full stop after “…17% adolescents). New sentence “Pilot…”
- Line 644 – adding the age range of the female interviewers could be an added value?
- Line 653 - comma after “ In addition to this…”
- Line 655-657- “one of the study aims…” – please clarify if it is really part of aims or objectives of the study (same as in line 670).

**Limitations:**

1) Unequal number of participants across categories (parents of children with health problems vs healthy children). In a qualitative study, having an unequal number of participants across categories (e.g., gender, profession, age groups, etc.) can introduce bias or limit the diversity of perspectives. When writing this as a limitation, the goal is to acknowledge the imbalance while demonstrating awareness of its implications for the study’s findings or transferability, it may reduce the study’s ability to fully explore variations between groups. Future research with more balanced sampling could offer a more comprehensive understanding.
2) Two different recruitment approaches – didn’t also add on a bias on the study?
3) Check that it is stated as a limitation in the text: line 236. *We acknowledge the potential bias from using interviewer-led mode”*
4) Given the strong emotional and ethical aspects of this study, it would be valuable to examine how the participants' consent form was formulated.
5) Ethical considerations could be stated as a reason for not sharing the video and interview materials (transcripts),
