# Supplementary material for: A qualitative study to understand public views on the relative value of health gains for children and young people in Australia compared to adults
Source: PLoS One. 2025 Oct 31;20(10):e0319227. doi: 10.1371/journal.pone.0319227 (PMC12578166; doi:10.1371/journal.pone.0319227)
Supplement: Attachment — Submitted filename: Response to Reviewer Comments.docx [file pone.0319227.s012.docx]

**Addressing Reviewer Comments**

*All changes made are shown in italics and underlined.

Dear Dr Paolo Landa,

I sincerely appreciate your thoughtful comments and feedback on our manuscript we are grateful for the time and effort you have dedicated to reviewing it.

**Reviewer #4**

Dear author,

I understand the importance of evaluating the personal rules involved in choices regarding investments in health programs by age.

1) In your article, I missed the addition of scientific studies on health outcomes related to the age of patients which should be also included to help decision-making. See, for example, articles on investments in health in early childhood. I suggest that you add some references to articles in this sense and that you mention the importance of such a source of decision-making in health.

**Response:** Thank you for your feedback. We have now incorporated this in the discussion section and added references.

“Seven study participants chose the younger age group because they felt children/adolescents should experience more in life and they should get the opportunity to experience all the things that adults (40 or 55 years) have experienced. This is consistent with the findings from Schweda et al. [32], who reported some participants felt that extending a person’s life was more important and legitimate if they are young because they could experience a more desirable life.

*Even though, in the PTO task we explicitly stated that the health gain was the same for both age groups, a few participants did think about the long term benefits a child may get compared to adults. This aligns with scientific evidence supporting long-term benefits of health investments in early childhood (Campbell et al, 2014).*

Participants also made their trade off in PTO questions by examining how being in a health condition would affect their families and society as well *- a finding also reported by* Schweda et al. [32].”

Reference: Campbell F, Conti G, Heckman JJ, Moon SH, Pinto R, Pungello E, et al. Early childhood investments substantially boost adult health. Science. 2014;343(6178):1478-85.

**Reviewer #5**

Authors addressed all comments raised by the reviewer in beautiful manner. Now this manuscript is ready for publication.

**Response:** Thank you for your positive feedback on our revised manuscript. We are grateful for your time and effort in reviewing our work.

**Reviewer #6**

TITLE

1) The title precisely reflects the subject of the study; however, it is academically advantageous to consider substituting the term "people" with "public."

“A qualitative study to understand **public** views on the relative value of health gains for children and young people compared to adults”

**Response:** Thank you for raising this point. Reviewer #8 suggested to mention in which country the study was conducted.

Therefore, we have now revised the title as “A qualitative study to understand *public* views on the relative value of health gains for children and young people *in Australia* compared to adults”

ABSTRACT

2) Methods: Concise overview. Nonetheless, I suggest the inclusion of the following statement at the last line of the methods used paragraph: “**Thematic analysis was employed to identify fundamental reasoning patterns.**”

**Response:** Thank you for the comment. We have now added the sentence into the methods section.

“Methods: Think-aloud, semi-structured interviews were conducted with Australian adolescents (n=7), non-parents (n=11), parents with healthy children (n=8) and parents of children with health conditions (n=15). Participants completed Person Trade-Off (PTO) and attitudinal questions about resource allocation for improvements in life extension, mental health, mobility, and pain/discomfort choosing between interventions for adults (ages 40 or 55) and younger people (ages one month to 24). *Thematic analysis was employed to identify fundamental reasoning patterns.*”

INTRODUCTION

The introduction is thorough and based in robust research. However, Structural enhancements are necessary to improve the clarity and scholarly tone, such as:

3) Lines (108 –128), (129-142), The paragraphs are too dense and long, consider **break them into clear paragraphs**.

**Response:** Thank you for raising this point. We agree that dividing the paragraphs will enhance the readability. The section now reads as follows:

“Differences in the age-related social values of QALYs are usually based on one of two rationales, efficiency considerations or equity/fairness considerations [3]. Efficiency considerations draw on the relationship between economic productivity and age, with consideration for broader social contributions to vary by age [4]. Fairness considerations draw on the fair innings argument. This was first presented by Harris [5] who argues that everyone should be given an equal opportunity to reach a normal span of years. According to this view, those who have not had their fair innings *need* to be prioritized. Williams [6] extended the fair innings argument to incorporate a person’s quality of life as well as length of life. Tsuchiya et al. [7] reported that people favour giving priority to younger people based on the fair innings argument, and to older people based on efficiency considerations. Schwappach [8] suggested the social value of a QALY may vary according to patients’ characteristics i.e., age, social role, lifestyle or severity of illness.

Existing studies have explored whether age should be considered as a criterion in allocating healthcare resources but the evidence on preference relating to age-related prioritization is mixed. Some studies identified a willingness to prioritize all children aged below 15 [9]. Other studies suggest support for prioritising children but only provide evidence for those *older than 5 years*. For example, Richardson et al. [10] reported age weights for ages from 5 to 70. They reported a preference for age 5, 10, 15 and 20 for life extension and age 5, 10, 15 for quality of life improvements. Petrou et al. [11] reported relative age weights from a person trade-off (PTO) study and identified a preference for prioritizing the younger age for life extending treatments. In contrast, there are also studies which found participants prioritize adults aged 40 and 70 years over children aged 10 years [12]. Some studies produce findings suggesting everyone should be equally treated [13]. A few qualitative studies analyzed people’s views on prioritizing treatments for children compared to adults. Aidem [14] reported that policy makers believe healthcare needs to be prioritized based on efficiency and equity. Kuder and Roeder [15] reported that people believe patients should not be treated differently based on their age.

A recent systematic review synthesised international evidence on the relative social value of health gains for children (<18 years) and those of adults [16]. The review found evidence that the public were willing to prioritize children’s health gains over adult’s health gains. However, the review identified variations in results (1) based on the study methodology. *For* example the review identified differences in results based on the type of question i.e., attitudinal questions [17, 18] compared to choice based numerical questions [19], (2) across different perspectives the study questions were framed i.e., prioritization within the family, or as a citizen or adopting a decision maker perspective, (3) based on the age of the child, (4) based on participant characteristics such as age, gender, parental status and (5) based on whether the health gain referred to extensions of length of life or improvements in quality of life.

One of the limitations identified in this review was the limited number of studies that explored the rationale behind participant choices. *Tsuchiya [20] is a rare example*. Therefore, further research is needed to understand what drives individuals’ responses to preference elicitation questions in which age of the recipient of health gain differs. Qualitative work can help interpret such variations in findings, by providing an understanding of the underlying reasons, and insights into participants’ thinking patterns and principles when responding to different types of questions [14, 15].”

4) Line 113, those who have not had their fair innings needs to be prioritized. Change needs to “**need**”

“According to this view, those who have not had their fair innings *need* to be prioritized.”

5) Line 120, consider changing >5 years to “**older than 5 years**” for more academic tone.

“Other studies suggest support for prioritising children but only provide evidence for those *older than 5 years*”

METHODS

Demonstrate methodological transparency, provide a strong rationale for recruitment, and offer clear justification for the selections. Nevertheless, it requires slight modifications for improved clarity, formatting, and organization.

6) Recruitment and Participants: Lines (164-179) and (180-192) consist of extensive paragraphs; **please divide them into shorter paragraphs**.

**Response:** Thank you for your suggestion on improving readability of the paper. We have now divided this paragraph into sections and also have made a change to the sentence involving adolescents to highlight reviewer #8 suggestions.

“Existing literature has found that attitudes towards prioritizing child health gains vary depending on the age and parenthood status of the participants [16]. In addition to age and parenthood, we hypothesized that parent’s experiences of child ill health may be relevant. Therefore, our recruitment ensured coverage of non-parents, parents of healthy children, and parents of children with a health condition across different age groups.

We also included older adolescents (aged 16 -18 years) at the request of the QUOKKA’s Decision Makers’ Panel. The recent review [16] identified that there *was a lack of* qualitative studies *specifically examining adolescent’s perspective on age-based healthcare prioritization,* yet studies have shown it is feasible for adolescents to value health states [24] which involves tasks with a similar level of cognitive and emotional difficulty to PTO questions. Adolescents (aged 16-18) coped well during the pilot interviews, which are described at length elsewhere [22].

We anticipated saturation would be reached at a sample of 40 interviews on the basis of Ritchie et al. [25] who suggested that studies involving a very diverse population might require an increased sample size, but a sample of fewer than 50 will be adequate for individual interviews. This is further supported by Hennink et al. [26] who suggested saturation would *be achieved* between 16-24 interviews and Guest et al. [27] who found saturation occurred within 12 interviews. Consideration of saturation adopted the approach by Guest et al. [27] which “refers to the point during data analysis at which incoming data points (interviews) produce little or no new useful information relative to the study objectives” [27].”

7) Survey Design: The paragraph is extensive, consisting of lines 198-210. It is desirable to **divide it into two paragraphs**.

**Response:** Thank you for your suggestion. The survey design section now reads as follows:

“The survey included six components, including consent and introduction video, seven PTO questions, feedback questions on comprehension, questions asking for reasons for PTO answers, attitudinal questions on health prioritization and demographic questions. Further details of the tasks and questions are provided in the published protocol study [22]. The seven PTO tasks involved different aspects of health improvement, including life extension (2 or 5 years), and improvements in aspects of quality of life (mental health, mobility and pain or discomfort). Examples are shown in Figure 1 and 2. There were four life extension questions and three quality of life questions.

Participants were asked to make choices between pairs of interventions, one impacting an adult group (either 40 or 55 years old) and the other younger group consisting of 13 age categories (one month, even number of years between 1 year and 24 years old). One of the life extension questions (applied to all participants) compared young people to other young people of a different age as part of a chaining test for the quantitative study. The ages used in the PTO questions was randomly selected, however for the final four interviews an age < 4 years was chosen for the younger age group to further explore findings arising from the analysis of the main survey data. Half of the sample were randomly given the option to select ‘no preference’ (unforced-arm) between the two hypothetical health programs in the seven PTO tasks, the other half were always required to choose between Program A or B (forced-arm). Interviewer prompts included discussion of their likely answer if they had seen the alternative presentation (i.e., when a choice between two programs was forced).”

8) Data Collection: The paragraphs lines (218-227) and (233-253) are extensive; therefore, it is necessary to **divide them into separate paragraphs**.

**Response:** Thank you for your suggestion. The data collection section now reads as follows:

- Line 218-227

“The interviews were conducted by three female interviewers (AD, TPE and CB). AD had experience in conducting quantitative interviews with adults and received training at the start of the study. TPE and CB were experienced in conducting qualitative interviews. Regular debriefs following the interviews were conducted among the three interviewers. Discussions included reflecting on initial interviews, re-listening to interview recordings and evaluating interviewer prompts.

We conducted an initial two pilot interviews with a convenience sample (known to the interviewers) to confirm interview prompts and processes; this data was not included in the analysis. The first six interviews were treated as a second pilot. The pilot interviews were rewatched and discussed including a reflection on the whether the prompts led to discussion which addressed the study questions. As no major changes were made to the interview prompts at this stage this pilot data is included in the main sample. Detailed information about the pilot and development of the survey prompts are described in the protocol paper [22].”

- Line 233-253

“At the start of the interview the interviewer explained how to participate in a think-aloud interview by demonstrating an example question. During the interview if the participants became quiet, they were encouraged to think-aloud and to explain why they had made their choice. The interviewers then completed the survey on behalf of the participants.

We acknowledge the potential bias from using interviewer-led mode. However, interviewer-led mode could help improve participant’s comprehension of the survey tasks and facilitate responses. We had made several efforts to mitigate potential biases, first, using neutral interview prompts (e.g., “I would like you to talk me through your thought process – just saying whatever is coming into your head. There are no right or wrong answers or thoughts here”) to ask survey questions, second, the survey began with an introduction video which talked through an example PTO question. In this video it was mentioned that participants could have views favoring young people or favoring adults.

Participants then answered seven PTO questions and three attitudinal questions and were invited to ‘think-aloud’ while they answered and to provide reasons behind their responses. They were also asked semi-structured questions to further probe their thinking and the reasons for their answers by answering feedback questions. The interviewers probed to explore differences in PTO responses between the different types of health gain. Where PTO responses appeared to give different preferences to attitudinal responses the interviewer asked for an explanation. If appropriate, the interviewer referred to other participants who had given apparently inconsistent views on these questions to ensure the participant did not feel their responses were being challenged. To explore the strength and robustness of the participants’ views the interviewer also raised that some people had given us very different opinions to their own (e.g. prioritizing children over adults or prioritizing adults over children) and asked how they felt about this. lnterviews were video recorded and transcribed intelligent verbatim using automated transcription which was checked by interviewers using the video.”

RESULTS

9) Participants section: It is well written; however, **please divide it into paragraphs** for enhanced readability.

**Response:** Thank you for your suggestion. The participants section now reads as follows:

“Recruitment took place between 27th March 2023 to 20th July 2023 and was completed after achieving thematic saturation. After 10 interviews were completed for each of the recruitment approaches (total 20), the researchers discussed the interviews to see whether new information could be gathered. Since new information was being generated, we continued conducting interviews. Our approach to determine saturation was based on thematic saturation and we considered saturation to be achieved when no new information relevant to the study *objectives* emerged. Once the researchers analyzed the last three interviews from the P-MIC sample, they decided that no new information relevant to study objectives was being generated and that we achieved thematic saturation. Data saturation was reached after the 38th interview.

A total of 41 participants were interviewed; 26 were recruited through CRNRstone and 15 from the P-MIC sample-frame. Of the 41 participants 56% were female and their mean age was 37 years. Of the 26 participants recruited through the CRNRstone sample 11 were female. Of the 26, 7 were adolescents, 11 were adults with no children, 2 were parents with children with a health condition and 6 were parents with healthy children. Of the 15 participants recruited from the P-MIC sample, 12 were female and of the 15, 13 were parents with children with a health condition and 2 were parents with healthy children. Regarding employment status in the CRNRstone sample, 12 participants were employed/ self-employed, 9 were students, and 5 were retired. In the P-MIC sample, 12 were employed/ self-employed, 1 was on maternity leave and 2 were not working. The background characteristics of study participants are provided in supplementary material (S1 and S2 tables).”

Qualitative Results: it is well organized and comprehensive, but consider this:

10) rewrite the sentence “Another participant chose the 40-year-olds over the 8-year-olds” in the line 333, for more academic tone **“participants aged 40 over those aged 8”**

“Another participant *prioritized individuals aged* 40 over *those aged* 8 by saying…..”

11) line 337 change the statement “people who are younger have to walk a lot more than someone who's 40” to **“Younger participants were viewed as more reliant on physical mobility, compared to those who are 40 years old”**

**Response:** Thank you for your suggestion. However, this is a quote extracted from the transcript, therefore we cannot change it.

12) Theme 5: Provide a short analytical statement elucidating the reason for this and why it happened for instance, **“Despite the guidance, participants struggled to disengage from real-world connections to chronic illness, illustrating the psychological challenge of ‘bracketing’ prolonged considerations.”**

**Response:** Thank you for your suggestion. We have now revised this section with a statement as follows:

“The PTO questions instructed participants to assume that the overall costs, carer’s health and wellbeing and any loss of income of carers or patients are the same in both the programs and there is no long-term health impact. Although most of the participants adhered to these assumptions, there were participants who understood that they were asked not to consider long term effects but did think about how the health conditions might have a long-term impact in the future. *Even with explicit instructions, participants found it difficult to detach from their understanding of real-world examples of long-term health impacts, illustrating the challenge in temporarily setting aside deeply ingrained health-related perceptions and experiences”*

DISCUSSION

This discussion is substantial and effective, but it can be enhanced through refinement in:

13) lines (535-554) and (637-662), are lengthy, **please divide them into paragraphs**.

**Response:** Thank you for your suggestion. This section now reads as follows:

Line 535-554:

“The findings from this qualitative study, which is a part of the wider PTO study [23], will enrich our understanding and interpretation of the quantitative components. The quantitative analysis of the mixed method PTO reported (1) children aged one month to 2 years old are given less weight than 40 or 55-year-olds in averting mental health problems, (2) all children (including one month old to 2 years) are given more weight than adults (aged 40 and 55) when considering averting pain, (3) when the older age group is 55-year-olds (rather than 40 years old) participants are more likely to prioritize the younger age group and (4) the youngest participants are more likely to prioritize the younger age in PTO tradeoffs.

From the qualitative interviews, we found potential reasons behind the quantitative patterns reported by [23]. We found that participants were unable to imagine a child aged one month old – 2 years old could be diagnosed with a mental health condition. Second, participants were emotional when thinking about a child being in pain and expressed not wanting to see them suffer. Similarly, Powell et al. [31] reported that adults believed being in pain or discomfort could be more challenging to a 10-year-old compared to adults. Third, the participants were more likely to draw upon ‘fair innings’ justification [6] for prioritizing adults when the adults were *55-year-olds*, as they felt had already experienced life. Finally, it was evident that compared to adults, adolescents in our study considered the health of children or adolescents (mostly patients aged >10 years) as more important than that of adults and provided clear rationales; for example, ability to perform usual activities, to cope, and to meet societal expectations. An interesting finding was that younger people focused on the things that young people do (which are closer to their own experience), for example going to school, doing sports and travelling. They also thought that older people have already experienced life. Older participants focused more on the importance of adults’ contribution to the family, work and society.”

Line 637-662:

“Nevertheless, this current study also has some limitations. The recruitment methods in our study may have introduced selection bias. Since the participants were recruited from a pool of participants willing to undertake qualitative research, this may potentially affect the generalizability of our findings. It is important to mention that this study was conducted involving participants from Australia, which might not be directly applicable to other social norms and values of other countries such as Asian, African countries. For example, existing literature indicates financial support, care support is provided by adult children for the wellbeing of older adults in China [38, 39]. These differences in social values may impact healthcare prioritization decisions.

All interviewers were female, which may lead to potential response biases if some participants might have answered differently in relation prioritizing health care for children compared to adults. McNay [40] reported that gender has the most impact in determining the interactions between the interviewer and participant and the emotions of the participants. Male participants are also considered to develop their responses according to the gender of the interviewer [41]. Another potential limitation of this study is the lack of formal researcher reflexivity during the study, such as keeping a reflexive journal throughout. Informal discussions and reflections considered that the three interviewers were economists which might have influenced their perspective on rational trade-offs in PTO choices. While the interviewers used survey prompts described elsewhere [22] to explain that there are no right or wrong views, it is unclear whether this could mitigate potential biases this background brought to the interviews.

In addition to this there was a lack of depth of discussion relating to scrutinizing and challenging people’s views. These discussions remained light touch given the sensitive nature of the topic, particularly for parents with children with health conditions. One of the study objectives was to explore whether the participants think the PTO questions were able to identify the relative weight they would give to improve child versus adult health. However, the survey prompts did not generate much discussion on this meta level concept and issues relating to this objective were not identified in the data. The study also aimed to understand the differences in responses to methods.

The study suggests attitudinal questions encourage people to think about fairness of access and need for additional healthcare funding. However, the direct comparison was hindered by the broad age categories used in the attitudinal questions given people’s nonlinear preferences towards prioritizing across age groups.”

14) Line 566-571 showing your novel contribution to the literature and the readers need to see this, so please add a statement that clarify this in the **line 571** such as “**To our knowledge, this is the first study to document………”**

**Response:** Thank you for your suggestion. We have now added a sentence to reflect the novel contribution from this study.

“One of the interesting findings in this study was observing how *most participants* reevaluate the size of the gain in the PTO question depending on the age of the group and selected the group with the largest gain. This occurred in both the HRQoL and life expectancy questions. They considered what the patient would experience during their current age or considered what age they would be before and after treatments to understand what experiences they might miss if they do not get the treatment. This has not been reported in any previous PTO study. *To our knowledge, this is the first study to document how participants actively reevaluate and contextualize health gains based on the age of the recipient of the treatment in PTO questions.”*

15) Line 542, change the informal statements please “unable to imagine” to **“had difficulty conceptualizing**

**Response:** Thank you for your suggestion. We have now made this change.

“We found that participants *had difficulty conceptualizing* a child aged one month old – 2 years old could be diagnosed with a mental health condition.”

CONCLUSION

It offers clear alignment with study objectives, acknowledges cognitive and emotional complexity, and is relevant to policy and methodology. However, please consider some amendments such as:

16) Line 674, These interpretations were reasoned by factors such as… change to These interpretations were **influenced** by factors such as… (for an elevated research tone).

“These interpretations were *influenced* by factors such as the size of the gain for each age group based on their ability to perform activities, their ability to cope or understand and what they might experience if they live.”

17) Replace informal language with academic ones such as line 671 “drawing on emotional reaction” to **“triggering emotional response”**, and line 676 “what they might experience if they live” to **“anticipated life experiences”**

Line 671:

“(1) Participants interpretation of PTO questions involved *both cognitive thought processes and emotional considerations. These included reflection on personal experiences, analysis of fairness in life outcomes and processing of emotional responses to the age group presented.”*

Line 676:

“These interpretations were *influenced* by factors such as the size of the gain for each age group based on their ability to perform activities, their ability to cope or understand and *anticipated life experiences*”

18) Add word **“Additionally”** before (4) When subjected to alternative viewpoints… (To enhance the coherence between sentences for improved readability).

“*Additionally*, 4) when subjected to alternative viewpoints many demonstrated that they accept each other’s opinions.”

19) The conclusion indicates that **four out of five aims** were addressed; however, it does not provide **a brief explanation** for the aim that was not addressed. A brief mention would enhance transparency.

**Response:** Thank you for raising this point, this is already addressed in the discussion section however we have added a sentence to the conclusion around future research:

“In addition to this there was a lack of depth of discussion relating to scrutinizing and challenging people’s views. These discussions remained light touch given the sensitive nature of the topic, particularly for parents with children with health conditions. One of the study *objectives* was to explore whether the participants think the PTO questions were able to identify the relative weight they would give to improve child versus adult health. However, the survey prompts did not generate much discussion on this meta level concept and issues relating to this *objective* were not identified in the data. The study also aimed to understand the differences in responses to methods.”

Conclusion:

*“While this study addresses four of its five objectives, it is important to note that one objective remains unexplored. Specifically, future research needs to address whether the participants believe that PTO questions effectively capture their relative weighting of child vs adult health improvements. This study did not generate sufficient discussion on this meta-level concept to allow us to draw conclusions.”*

20) The final sentence suggests practical relevance; however, the conclusion would be enhanced by adding a **strong closing statement** regarding the implications of the findings for healthcare policymakers, PTO methodology, or valuation research. Please consider adding this.

**Response:** Thank you for your suggestion to enhance our conclusion. We have now added a closing statement.

“This study explored the views of Australian adolescents and adults on how they feel about valuing health gains differently based on age and for different types of health gains. Our study addresses four of the five *objectives* set out (1) Participants interpretation of PTO questions involved *both cognitive thought processes and emotional considerations. These included reflection on personal experiences, analysis of fairness in life outcomes and processing of emotional responses to the age group presented*. (2) Differences in PTO choices between prioritizing children or adults were largely driven by the way the participants interpret the impact of the HRQoL state or additional years of life. These interpretations were *influenced* by factors such as the size of the gain for each age group based on their ability to perform activities, their ability to cope or understand and *anticipated life experiences*. (3) Inconsistencies between PTO and attitudinal questions were largely driven by participants consideration on fairness and access to healthcare. Additionally, (4) when subjected to alternative viewpoints many demonstrated that they accept each other’s opinions. These qualitative insights help to inform the development of different approaches in healthcare resource allocation highlighting the importance of involving a diverse range of participants with varying views. *Furthermore, the complex cognitive processes and varied interpretations revealed in the study highlight the need for more nuanced approaches to PTO methods. While this study addresses four of its five objectives, it is important to note that one objective remains unexplored. Specifically, future research needs to address whether the participants believe that PTO questions effectively capture their relative weighting of child vs adult health improvements. This study did not generate sufficient discussion on this meta-level concept to allow us to draw conclusions.*

**Reviewer #7**

Thank you for the invitation to review. The authors have appropriately revised their manuscript in accordance with the previous reviewers' requirements. I believe the manuscript now meets the standards for publication in PLOS ONE, and I recommend its acceptance.

**Response:** Thank you for your thoughtful feedback. We appreciate for taking the time to review our manuscript and for your positive recommendation.

**Reviewer #8**

I have some few observations on the study title which to be address by the authors to facilitate reading and better understanding by the authors.

1) A qualitative study to understand some selected individual's views on the relative value of health gains for children and young people in..... (here the name of the exact place where the study took place must be mentioned.) in Australia compared to Adults .....(which year was the study conducted )?

**Response:** Thank you for your suggestion to include the location, we have now revised the title. Regarding the specific location within Australia, we have chosen not to include because while the PMIC sample is indeed focused on people from Victoria, the CRNRSTONE sample includes participants from multiple states and territories, including New South Whale, Vicstoria, Queensland, Tasmania, South Australia, Western Australia and Australian Capital Territory.

“A qualitative study to understand *public* views on the relative value of health gains for children and young people *in Australia* compared to adults”

Regarding the inclusion of the study year, in qualitative research of this nature it is not standard practice to include the year in the title since the findings often have relevance beyond the specific timeframe of data collection.

However, we have included the year in the Abstract.

“Think-aloud, semi-structured interviews were conducted with Australian adolescents (n=7), non-parents (n=11), parents with healthy children (n=8) and parents of children with health conditions (n=15) *over a period of four months (27th March 2023 - 20th July 2023)*”

2) Methods

Under line 181 which says that adolescents and adults without children -Can the authors describe and elaborate further on what they meant by without children whether biological or adopted children or had children but are not staying with them (empty net syndrome)

**Response:** Thank you for your question. In our study we asked participants a simple yes or no question about whether they have (or ever had) children. We did not collect more detailed information about the nature of their parental status, such as whether the children were biological, adopted, or living elsewhere.

3) Pre-testing of questionnaire,

were the questionnaire pre-tested and where were they pre-tested (pretesting of questionnaire can’t take place in the study area).

**Response:** Thank you for your question. Our pre-testing process consisted of two stages:

1. Initial Pilot: We conducted two pilot interviews with a convenience sample (individuals known to the interviewers). These interviews were specifically designed to test and refine our interview prompts and processes. The data from these initial pilot interviews was not included in our final analysis.

2. Second Pilot: The first six interviews of our main study were treated as a second pilot. We closely monitored these interviews to ensure that our refined prompts and processes were effective in addressing our research questions. Our intention was to thoroughly assess these interviews and exclude the data if any issues arose.

It's important to note that the second pilot was conducted within the study area, as it was part of our main data collection process. However, we were prepared to exclude this data if necessary. As no major changes were required to the interview prompts following this assessment, we included these six interviews in our main sample.

The initial pilot interviews were conducted with individuals outside of our main study sample, adhering to the principle that pre-testing should not take place in the study area.

We incorporated these changes in the paper as below.

“We conducted *a two-stage pilot process to refine our questionnaire and interview approach.* *Initially,* two pilot interviews *were conducted* with a convenience sample (known to the interviewers) to confirm interview prompts and processes; this data was not included in the analysis. *Subsequently*, the first six interviews were treated as a second pilot. *The intention was to thoroughly assess these interviews and exclude the data if any issues arose.* The pilot interviews were rewatched and discussed including a reflection on the whether the prompts led to discussion which addressed the study questions. As no major changes were made to the interview prompts at this stage this pilot data is included in the main sample. Detailed information about the pilot and development of the survey prompts are described in the protocol paper [22].”

4) In line 631 there is an incomplete statement

some strengths of the study were that we included an adolescent sample to address the research gap identified review by...this part of the statement must be concluded with purpose and meaning.

**Response:** Thank you for your suggestion, we have now amended this sentence to make it more meaningful.

“Some strengths of this study were that we included an adolescent sample to address the research gap identified through the review [16]. *The review highlighted a lack of qualitative studies specifically examining adolescent’s perspective on age-based healthcare prioritization. By including adolescents in our study, we have contributed to filling this gap in the literature and provided valuable insights into how younger individuals view healthcare prioritization across different age groups.*”

We have also adjusted this in the methods- recruitment and participants section:

“We also included older adolescents (aged 16 -18 years) at the request of the QUOKKA’s Decision Makers’ Panel. The recent review [16] identified that there *was a lack of qualitative studies specifically examining adolescent’s perspective on age-based healthcare prioritization*, yet studies have shown it is feasible for adolescents to value health states [24] which involves tasks with a similar level of cognitive and emotional difficulty to PTO questions.

5) Conclusion.

Since the study was not conducted in the entire Australia the Authors can’t conclude that the study explored the views of Australians, but a statement can be made to cover the exact geographical location where the study took place.

**Response:** Thank you for your suggestion. However, while the PMIC sample is focused on people from Victoria, the CRNRSTONE sample include participants from NSW, Victoria, Queensland, Tasmania, South Australia, Western Australia and ACT. We believe this broad geographical representation enhances the validity of our findings as reflective of Australian views more generally.

We have now expanded the strengths section to include information about the geographical representation of the sample.

“Some strengths of this study were that we included an adolescent sample to address the research gap identified through the review [16]. *The review highlighted a lack of qualitative studies specifically examining adolescent’s perspective on age-based healthcare prioritization. By including adolescents in our study, we have contributed to filling this gap in the literature and provided valuable insights into how younger individuals view healthcare prioritization across different age groups.*

The study also had a large sample size *and broad geographical representation. While the PMIC sample focused on participants from Victoria, the CRNRSTONE sample included participants from New South Wales, Victoria, Queensland, Tasmania, South Australia, Western Australia and Australian Capital Territory. Furthermore, the study demonstrated breadth of coverage in terms of participant characteristics (56% females, 76% born in Australia, 17% adolescents).*

*Pilot* interviews were carried out and the study was a combination of think-aloud and probing *techniques*. Think-aloud interviews helped us to better understand the participants’ thought *processes*. Our study was conducted by three interviewers who could facilitate discussion and viewpoint diversity among the participants.”

6) Check line 630 where you talked about addressing four of the five aims set out, can the authors explain why the 5th aim was not address in the study or why it is not mentioned in the write-up

**Response:** Thank you for raising this point, this is already addressed in the discussion and conclusion sections:

Discussion-

“In addition to this there was a lack of depth of discussion relating to scrutinizing and challenging people’s views. These discussions remained light touch given the sensitive nature of the topic, particularly for parents with children with health conditions. One of the study *objectives* was to explore whether the participants think the PTO questions were able to identify the relative weight they would give to improve child versus adult health. However, the survey prompts did not generate much discussion on this meta level concept and issues relating to this *objective* were not identified in the data. The study also aimed to understand the differences in responses to methods.”

Conclusion:

*“While this study addresses four of its five objectives, it is important to note that one objective remains unexplored. Specifically, future research needs to address whether the participants believe that PTO questions effectively capture their relative weighting of child vs adult health improvements. This study did not generate sufficient discussion on this meta-level concept to allow us to draw conclusions.”*

**Reviewer #9**

I consider this to be an interesting article, and one that warrants further work on the subject. I believe that the authors have taken the reviewers' corrections seriously, which has helped them to improve their manuscript.

**Response:** Thank you for your feedback. We are pleased that you find our article interesting and that you see potential for further work in this area.

**Reviewer #11**

This is a strong qualitative study that provides nuanced insights into societal views on age-related healthcare prioritization. With minor clarifications and refinements as suggested, the manuscript will make a valuable contribution to the literature on health economics, ethics, and resource allocation.

**Response:** Thank you for your positive feedback on the paper.

**Reviewer #12**

1) Clarify Terminology Consistently: Ensure consistent use of terms like “children,” “adolescents,” and “young people” throughout the manuscript and figures to avoid confusion.

**Response:** Thank you for flagging this. This was raised by one of the reviewers in the last round.

- Adolescents (participant): 16-18 years
- Young people (question): 0-24 years
- Children: (generally referring to individuals under 18 years of age, unless otherwise specified)

We have now mentioned these definitions added at the first point in the paper.

- “We also included older adolescents (aged 16 -18 years)…”
- “PTO study to estimate the average relative weight for health gains for children and young people (aged 0 to 24 years)..)”
- “A recent systematic review synthesized international evidence on the relative social value of health gains for children *(generally referring to individuals under 18 years of age, unless otherwise specified)* and those of adults..”

2) Improve Figure Quality: Figures 1 and 2 (PTO examples) and Figure 3 (Framework Analysis) should be provided in high-resolution, publication-ready format. Ensure all text is legible.

**Response:** Thank you for raising this. We apologize for the lack of clarity in figure 1, figure 2 and figure 3. We have now amended this.

3) Reflexivity and Positionality: While limitations are acknowledged, consider briefly expanding on how the interviewers’ backgrounds as economists may have influenced data interpretation, not just data collection.

**Response:** Thank for your suggestion. We have now addressed this in more detail in the limitations section.

……“Another potential limitation of this study is the lack of formal researcher reflexivity during the study, such as keeping a reflexive journal throughout. Informal discussions and reflections *acknowledged* that the three interviewers’ *backgrounds in health economics* might have influenced their perspective on rational trade-offs in PTO choices. While the interviewers used *standardized* survey prompts described elsewhere [22] to explain that there are no right or wrong views, *we acknowledge that* this *may not have fully* *mitigate*d potential biases *occurring from* their *professional* background. *However, in the data interpretation approach, we ensured to consider a range of factors beyond economic optimizations. These included attention to participants’ emotional responses, personal experiences and cultural influences.”*

4) COREQ Checklist: Ensure the COREQ checklist (S4 Table) is complete and accurately reflects the study’s reporting.

**Response:** Thank you we have now amended the COREQ checklist. The COREQ checklist page numbers refer to the version of the manuscript with figures inserted.

5) Reference Formatting: Some references (e.g., [23]) are still marked as “unpublished manuscript.” If now published, update citation details accordingly.

**Response:** Thank you, the paper is yet to be published.

**Reviewer #13**

Congratulations to the author for conducting qualitative research on a very pertinent topic that will guide the policy makers for decision making.

Thank you for taking your time to review the manuscript and thank you for your feedback.

However, there are few suggestions to improve the quality of the research paper.

1) Please mention the type of qualitative research conducted, what were the strategies used to maintain the rigor of the qualitative study, what method/framework was used for the qualitative data analysis?

**Response:** Thank you for your suggestion. We have now amended the section ‘data analysis’ as below:

*Qualitative Research Approach and Data Analysis*

*“A thematic analysis was employed to identify the fundamental reasoning patterns in participants’ responses. This approach allowed us to identify, analyse and report themes within the data. Our approach incorporated* a framework analysis approach [30] *as described in Figure 3* for data analysis. Coding adopted both deductive (drawing upon the structure of the PTO survey and the study *objectives*) and inductive approaches. The final stages of the analysis included examining the coded data according to key demographic characteristics including gender, parental status and whether parents had children with health issues. We then linked these qualitative results to the quantitative findings.”

2) Moreover, author need to describe in methodology, how many researchers were involved in data collection, how was the inter-coder's reliability data transparency checked?

**Response:** Thank you for your suggestion. These were mentioned in Figure 3, however we have now addressed these in the below section.

*“A thematic analysis was employed to identify the fundamental reasoning patterns in participants’ responses. This approach allowed us to identify, analyse and report themes within the data. Our approach incorporated* a framework analysis approach [30] *as described in Figure 3* for data analysis. *Three researchers (AD, TPE and CB) were involved in the data collection and analysis process. To ensure inter-coder reliability and data transparency we followed a detailed coding process. Initially, all three researchers independently coded two transcripts to highlight broad themes in the interviews. The researchers then met to discuss the initial coding, where similarities and differences were discussed, and changes were incorporated into the existing codes. A further five transcripts were double coded, and the researchers discussed the list of codes, adding extra codes during these discussions. One researcher (AD) then applied the final coding framework to all the transcripts.*

Coding adopted both deductive (drawing upon the structure of the PTO survey and the study *objectives*) and inductive approaches. The final stages of the analysis included examining the coded data according to key demographic characteristics including gender, parental status and whether parents had children with health issues. We then linked these qualitative results to the quantitative findings.”

3) Conclusion could be written aligned to objectives in a comprehensive manner.

**Response:** Thank you for your suggestion.

“This study explored the views of Australian adolescents and adults on how they feel about valuing health gains differently based on age and for different types of health gains. Our study addresses four of the five *objectives* set out, *providing valuable insights into the decision-making process of health-care prioritization.*

*First, we found that p*articipants interpretation of PTO questions involved *both cognitive thought processes and emotional considerations. These included reflection on personal experiences, analysis of fairness in life outcomes and processing of emotional responses to the age group presented. Second the study revealed that* differences in PTO choices between prioritizing children or adults were largely driven by the way the participants interpret the impact of the HRQoL state or additional years of life. These interpretations were *influenced* by factors such as the size of the gain for each age group based on their ability to perform activities, their ability to cope or understand and *anticipated life experiences*. *This highlights the importance of considering age-specific factors and the complexity of healthcare prioritization choices in healthcare decision making.*

*Third we identified* inconsistencies between PTO and attitudinal questions were largely driven by participants consideration on fairness and access to healthcare. Additionally, when subjected to alternative viewpoints many demonstrated that they accept each other’s opinions. These qualitative insights help to inform the development of different approaches in healthcare resource allocation highlighting the importance of involving a diverse range of participants with varying views. *Furthermore, the complex cognitive processes and varied interpretations revealed in the study, highlight the need for more nuanced approaches to PTO methods*

*“While this study addresses four of its five objectives, it is important to note that one objective remains unexplored. Specifically, future research needs to address whether the participants believe that PTO questions effectively capture their relative weighting of child vs adult health improvements. This study did not generate sufficient discussion on this meta-level concept to allow us to draw conclusions.”*

**Reviewer #14**

Concerns from previous reviewers have been addressed adequately, I have just raised minor concerns and add comments that could further improve the document. I support that the manuscript is accepted for publication.

Thank you for giving me the opportunity to review the article mentioned above.

Thank you for your thoughtful feedback and time in reviewing the manuscript.

I can see that previous reviewers have given fair remarks, to which I would like to add the following:

ABSTRACT

1) The objectives should be replaced by aim of the study, there is only 1 (if it was to be objective) mentioned, which reflects the aim of the study (line154) and the 5 objectives later (lines156-161)

**Response:** Thank you for your suggestion. We have amended this.

“The overall aim of this study is to provide evidence to decision makers in Australia on public opinion regarding the social value of child health gains relative to adult health gains. *To achieve this aim*, the qualitative analysis of interviews *focused on five objectives*: (1) understand how participants interpret and make choices in the PTO and what information they focus on, (2) explore whether they think the PTO questions can identify the relative weight they would give to improving child versus adult health, (3) understand participants’ reasons behind their preferences, (4) understand the reasons behind any inconsistencies between attitudinal questions and PTO responses and (5) understand how strongly views are held through subjecting participants’ opinions and responses to scrutiny, alternative views and disagreement.”

INTRODUCTION

2) Line 124 mentions “studies which found…”, yet there is only 1 reference (line125)

“In contrast, there *is also a study* which found participants prioritize adults aged 40 and 70 years over children aged 10 years [12].”

3) Line 148: “Key evidence…” – key should be in small letters

**Response:** Thank you for your suggestion. However, both 'Kids' and 'Key' should be capitalised, as they define the acronym “QUOKKA”.

“This current study is a part of the Q*U*ality Of life in *K*ids: Key evidence to strengthen decisions in Australia (QUOKKA) project…”

4) Recruitment and Participants: Line 167 – add the full range age of participants (16- xxx years), which is not the same as the target groups (e.g 2 years), because this latter group was not interviewed and didn’t contribute to the findings.

**Responses:** Thank you for your feedback. We would like to clarify that the age range of the actual participants is indeed provided in the supplementary table S1 of our manuscript. We have also added a sentence to the main text in the ‘results section’:

“A total of 41 participants were interviewed; 26 were recruited through CRNRstone and 15 from the P-MIC sample-frame. Of the 41 participants 56% were female and their mean age was 37 years *(range: 16 to 86 years).*”

Regarding the age group 2 years, it is not a target age group rather a hypothetical age group of the child in the PTO question.

5) Survey Design: Lines 202-203 (13 age categories) – please justify/explain choices for categories

**Responses:** Thank you for your feedback. We have now added a sentence to explain the reason behind the age choices.

“Participants were asked to make choices between pairs of interventions, one impacting an adult group (either 40 or 55 years old) and the other younger group consisting of 13 age categories (one month, even number of years between 1 year and 24 years old). *These age categories were chosen based on a recent systematic review [16] that identified evidence gaps in very young children (<5 years) and young adults (18-24 years) in studies exploring social value.*”

We have also adjusted first section of ‘survey design’:

“The survey included six components, including consent and introduction video, seven PTO questions, feedback questions on comprehension, questions asking for reasons for PTO answers, attitudinal questions on health prioritization and demographic questions. Further details of the tasks and questions are provided in the published protocol study [22] *and the paper discussing the quantitative results (Peasgood et al, 2025).* The seven PTO tasks involved different aspects of health improvement, including life extension (2 or 5 years), and improvements in aspects of quality of life (mental health, mobility and pain or discomfort). Examples are shown in Figure 1 and 2. There were four life extension questions and three quality of life questions.”

Reference: Peasgood T, U DSP, Chen G, Bailey C, Norman R, Viney R, et al. Are health gains to children and adolescents more important than health gains to adults? A person trade off study in Australia [Unpublished manuscript]. 2025.

Data Collection:

6) Line 234: example?

“At the start of the interview the interviewer explained how to participate in a think-aloud interview by demonstrating an example question *about choosing a cat versus a dog as a pet*.”

7) Line 241: that example could be provided (even if the video is not for public consumption)

**Responses:** Thank you for your feedback. We have now included this as supplementary S2_File and mentioned in the manuscript.

“…second, the survey began with an introduction video *(S2_File)* which talked through an example PTO question. In this video it was mentioned that participants could have views favoring young people or favoring adults.”

8) Line 250-251: please clarify…sentence is not clear

“To explore the strength and robustness of the participants’ views, the interviewer *presented contrasting opinions expressed by other participants* (e.g. prioritizing children over adults vice versa) and asked *how the current participant felt about these differing views*.”

9) Line 252: reads better as "Interviews were video-recorded and transcribed using automated intelligent verbatim transcription."

“Interviews were video-recorded and transcribed using *automated intelligent verbatim transcription*."

Data Analysis

10) Line 258: an important key demographic to consider was employment/unemployment – but in the manuscript, it is only mentioned later in the results section.

**Response:** Thank you for your feedback, we have now added this into the section.

“The final stages of the analysis included examining the coded data according to key demographic characteristics including gender, parental status, whether parents had children with health issues *and employment status*.”

11) Participants

Line 264 – recruitment was done over a period of four months (27th March- 20th July 2023)

“Recruitment *was completed* *over a period of four months (27th March 2023 - 20th July 2023)* after achieving thematic saturation.”

Qualitative results:

12) Table 1: 4.1 and 4.2 – small letters

4.1 Drawing on *o*wn *e*xperiences

4.2 Emotional *r*esponse

13) The authors should try as much as possible to replace words like many (lines 341, 523), few (line 352, 525), etc…by numbers/percentage (quantities) like you have done in other instances.

**Response:** Thank you for your feedback. We have now mentioned the number of participants, where it was mentioned as many, few participants.

- ‘Many’ was mentioned if it was more than 50% participants.
- ‘Some’ was mentioned if it was 25%-50% participants.
- ‘Few’ was mentioned if it was 15%-25% participants.
- ‘Very few’ was mentioned if it was less than 15% participants

Line 341: “Many participants *(27 participants)* used their perception of how the age group would cope with the condition as a way of prioritizing between groups.”

Line 523: “*Some* of the participants *(14 participants)* understood and accepted the viewpoints by saying..”

Line 352: “*Very few* parents with children with a health condition *(3 participants)* chose the adult age group considering that the child has sufficient support.”

Line 525: There were very few *(5 participants)* who opposed other participants’ viewpoints.

14) Line 390: What was program A -has it been defined/mentioned previously? (same as program B – line 397)

**Response:** Thank you for raising this. Under each quote where it says program A or B, it is mentioned in brackets which age group is program A.

15) Lines 572 and 580 – nice to see a number/percentage attached to the participants category, would like to see the same on line 579.

**Response:** Thank you, we have now added a number to this:

“Similarly, in our study we found *some* individuals (14 participants) who were reluctant to prioritize based on age.”

16) Line 633 – full stop after “…17% adolescents). New sentence “Pilot…”

**Response:** Thank you we have made this change.

17) Line 644 – adding the age range of the female interviewers could be an added value?

**Response:** Thank you we have now added the age range.

“The interviews were conducted by three female interviewers *aged between 27-53 years* (AD, TPE and CB).

18) Line 653 - comma after “ In addition to this…”

**Response:** Thank you we have made this change.

19) Line 655-657- “one of the study aims…” – please clarify if it is really part of aims or objectives of the study (same as in line 670).

**Response:** Thank you we have now amended this as: “One of the study *objectives* was to explore whether the participants think the PTO questions.”

LIMITATIONS

20) Unequal number of participants across categories (parents of children with health problems vs healthy children). In a qualitative study, having an unequal number of participants across categories (e.g., gender, profession, age groups, etc.) can introduce bias or limit the diversity of perspectives. When writing this as a limitation, the goal is to acknowledge the imbalance while demonstrating awareness of its implications for the study’s findings or transferability, it may reduce the study’s ability to fully explore variations between groups. Future research with more balanced sampling could offer a more comprehensive understanding.

**Response:** Thank you for your feedback we have now addressed this.

“Nevertheless, this current study also has some limitations. The recruitment methods in our study may have introduced selection bias. Since the participants were recruited from a pool of participants willing to undertake qualitative research, this may potentially affect the generalizability of our findings. *The study also includes unequal number of participants across categories (adolescents, parents, non-parents), which may potentially limit the diversity of perspectives captured.* It is important to mention that this study was conducted involving participants from Australia, which might not be directly applicable to other social norms and values of other countries such as Asian, African countries. For example, existing literature indicates financial support, care support is provided by adult children for the wellbeing of older adults in China [38, 39]. These differences in social values may impact healthcare prioritization decisions.

All interviewers were female, which may lead to potential response biases if some participants might have answered differently in relation prioritizing health care for children compared to adults. McNay [40] reported that gender has the most impact in determining the interactions between the interviewer and participant and the emotions of the participants. Male participants are also considered to develop their responses according to the gender of the interviewer [41]. Another potential limitation of this study is the lack of formal researcher reflexivity during the study, such as keeping a reflexive journal throughout. Informal discussions and reflections *acknowledged* that the three interviewers’ *backgrounds in health economics* might have influenced their perspective on rational trade-offs in PTO choices. While the interviewers used *standardized* survey prompts described elsewhere [22] to explain that there are no right or wrong views, *we acknowledge that* this *may not have fully* *mitigate*d potential biases *occurring from* their *professional* background. *However, in the data interpretation approach, we ensured to consider a range of factors beyond economic optimizations. These included attention to participants’ emotional responses, personal experiences and cultural influences.*

In addition to this there was a lack of depth of discussion relating to scrutinizing and challenging people’s views. These discussions remained light touch given the sensitive nature of the topic, particularly for parents with children with health conditions. One of the study *objectives* was to explore whether the participants think the PTO questions were able to identify the relative weight they would give to improve child versus adult health. However, the survey prompts did not generate much discussion on this meta level concept and issues relating to this objective were not identified in the data. The study also aimed to understand the differences in responses to methods.

The study suggests attitudinal questions encourage people to think about fairness of access and need for additional healthcare funding. However, the direct comparison was hindered by the broad age categories used in the attitudinal questions given people’s nonlinear preferences towards prioritizing across age groups.”

21) Two different recruitment approaches – didn’t also add on a bias on the study?

**Response:** Thank you for your feedback. We do not think this added a bias, the reason for including two recruitment approaches was to include a wide range of participants across different demographic characteristics.

22) Check that it is stated as a limitation in the text: line 236. We acknowledge the potential bias from using interviewer-led mode”

**Response:** Thank you for your suggestion. We have already included a section on biases that interviewers may bring.

“All interviewers were female, which may lead to potential response biases if some participants might have answered differently in relation prioritizing health care for children compared to adults. McNay [40] reported that gender has the most impact in determining the interactions between the interviewer and participant and the emotions of the participants. Male participants are also considered to develop their responses according to the gender of the interviewer [41]. Another potential limitation of this study is the lack of formal researcher reflexivity during the study, such as keeping a reflexive journal throughout. Informal discussions and reflections *acknowledged* that the three interviewers’ *backgrounds in health economics* might have influenced their perspective on rational trade-offs in PTO choices. While the interviewers used *standardized* survey prompts described elsewhere [22] to explain that there are no right or wrong views, *we acknowledge that* this *may not have fully* *mitigate*d potential biases *occurring from* their *professional* background. *However, in the data interpretation approach, we ensured to consider a range of factors beyond economic optimizations. These included attention to participants’ emotional responses, personal experiences and cultural influences.”*

23) Given the strong emotional and ethical aspects of this study, it would be valuable to examine how the participants' consent form was formulated.

**Response:** Thank you for your suggestion. We have now included the consent form as a supplementary document (S3_File).

24) Ethical considerations could be stated as a reason for not sharing the video and interview materials (transcripts),

**Response:** Thank you for your feedback. We have already included this in the data availability statement:

“The data in this study consists of quotes extracted from participant transcripts. These quotes are included within the manuscript and the Supporting Information (S3 Table). Consent from participants was obtained specifically for the use of these quotes in the study. In accordance with our ethical approval and participant consent agreements, we are not able to make the full interview transcripts publicly available. This restriction is in place to protect the privacy and confidentiality of our study participants. However, we are committed to the principles of open science and data sharing where ethically possible.”
